# Polydopamine Coating-Mediated Immobilization of BMP-2 on Polyethylene Terephthalate-Based Artificial Ligaments for Enhanced Bioactivity

**DOI:** 10.3389/fbioe.2021.749221

**Published:** 2021-11-16

**Authors:** Zhanrong Kang, Dejian Li, Chaoqin Shu, Jianhang Du, Bin Yu, Zhi Qian, Zeyuan Zhong, Xu Zhang, Baoqing Yu, Qikai Huang, Jianming Huang, Yufang Zhu, Chengqing Yi, Huifeng Ding

**Affiliations:** ^1^ Department of Orthopaedics, Shanghai Pudong Hospital, Fudan University Pudong Medical Center, Shanghai, China; ^2^ State Key Laboratory of High-Performance Ceramics and Superfine Microstructure, Shanghai Institute of Ceramics, Chinese Academy of Sciences, Shanghai, China; ^3^ School of Materials Science and Engineering, University of Shanghai for Science and Technology, Shanghai, China; ^4^ Department of Pain and Rehabilitation, Shanghai Public Health Clinical Center, Shanghai Medical School, Fudan University, Shanghai, China; ^5^ Shanghai Pudong New Area People’s Hospital, Shanghai, China

**Keywords:** artificial ligament, bioactivity, bone morphogenic proteins, graft-to-bone healing, polyethylene terephthalate, surface modification

## Abstract

**Background/objectives:** Polyethylene terephthalate (PET)-based artificial ligaments are one of the most commonly used grafts in anterior cruciate ligament (ACL) reconstruction surgery. However, the lack of favorable hydrophilicity and cell attachment for PET highly impeded its widespread application in clinical practice. Studies found that surface modification on PET materials could enhance the biocompatibility and bioactivity of PET ligaments. In this study, we immobilized bone morphogenetic protein-2 (BMP-2) on the surface of PET ligaments mediated by polydopamine (PDA) coating and investigated the bioactivation and graft-to-bone healing effect of the modified grafts *in vivo* and *in vitro*.

**Methods:** In this study, we prepared the PDA coating and subsequent BMP-2-immobilized PET artificial ligaments. Scanning electron microscopy (SEM) was used to analyze the morphological changes of the modified grafts. In addition, the surface wettability properties of the modified ligaments, amount of immobilized BMP 2, and the release of BMP-2 during a dynamic period up to 28 days were tested. Then, the attachment and proliferation of rat bone mesenchymal stem cells (rBMSCs) on grafts were examined by SEM and Cell Counting Kit-8 (CCK-8) assay, respectively. Alkaline phosphatase (ALP) assay, RT-PCR, and Alizarin Red S staining were performed to test the osteoinduction property. For *in vivo* experiments, an extra-articular graft-to-bone healing model in rabbits was established. At 8 weeks after surgery, biomechanical tests, micro-CT, and histological staining were performed on harvested samples.

**Results:** A surface morphological analysis verified the success of the PDA coating. The wettability of the PET artificial ligaments was improved, and more than 80% of BMP-2 stably remained on the graft surface for 28 days. The modified grafts could significantly enhance the proliferation, attachment, as well as expression of ALP and osteogenic-related genes, which demonstrated the favorable bioactivity of the grafts immobilized with BMP-2 *in vitro*. Moreover, the grafts immobilized with BMP-2 at a concentration of 138.4 ± 10.6 ng/cm^2^ could highly improve the biomechanical properties, bone regeneration, and healing between grafts and host bone after the implantation into the rabbits compared with the PDA-PET group or the PET group.

**Conclusion:** The immobilization of BMP-2 mediated by polydopamine coating on PET artificial ligament surface could enhance the compatibility and bioactivity of the scaffolds and the graft-to-bone healing *in vivo*.

## Introduction

Anterior cruciate ligament (ACL) injury, which may result in knee instability, secondary cartilage damage, and osteoarthritis (Shelbourne and Gray, 2000; Lohmander et al., 2007; Lidén et al., 2008), is a common injury among young adults and athletes (Zantop et al., 2008; Sofu et al., 2015). Currently, the gold-standard treatment for ACL rupture is ACL reconstruction surgery (Mascarenhas and MacDonald, 2008). Autograft tendon, allograft tendon, or artificial ligaments are available grafts for ACL reconstruction. Compared with allografts or autografts, artificial ligaments can not only meet the demand of stability and flexibility of knee joints, such as tension, flexion, and torsion (Huang et al., 2012; McDonald et al., 2021), but also overcome the drawbacks of donor site morbidity and reduce the incidence of disease transmission (Vaishya et al., 2015; Jia et al., 2017). A ligament advanced reinforcement system (LARS) artificial ligaments made by polyethylene terephthalate (PET) gained popularity in recent decades (Ahldén et al., 2009; Ichiba and Kishimoto, 2009; Karaoglu et al., 2009; Wang et al., 2015). A long-term follow-up on patients who have undergone ACL reconstruction with LARS ligaments reported satisfactory results in 84.6% cases and concluded that LARS ligaments were a safe and suitable option for ACL reconstruction (Parchi et al., 2018). However, the PET ligaments showed low cell affinity, which made it difficult for cell adhesion and led to inadequate interaction with host bone. Therefore, surface modification was introduced to improve the bioactivity of PET ligaments.

Studies have shown that surface modifications using chitosan, hydroxyapatite, graphene (Gustafsson et al., 2012; Vaquette et al., 2013; Li and Chen, 2015), VEGF (Lv et al., 2015), as well as laser could enhance the adhesion, proliferation, and osteogenesis differentiation of cells. Nevertheless, certain drawbacks, including difficult storage as well as uncontrolled delivery of growth factors, potential adverse effects *in vivo*, and laser modification (Li et al., 2017) may even impair the mechanical properties of artificial ligaments itself, hampering the application of these methods. Bone morphogenetic protein-2 (BMP-2), one of the transforming growth factors (TGFs), plays an important role in the initial stage of bone formation (Yao et al., 2020) and tissue regeneration (Poon et al., 2016). BMP-2 could promote the proliferation and osteogenic differentiation of bone mesenchymal stem cells (BMSCs) (Bayat et al., 2017; Yao et al., 2020) and stimulate the maturation of pre-osteoblast, consequently enhancing the secretion of bone-associated protein and mineralization (Zhang et al., 2017), and it is involved in bone metabolism as well (Rawadi et al., 2003; Chen et al., 2004; Cao and Chen, 2005). BMP-2 incorporated scaffolds that showed an excellent ability of osteoinduction and bone formation (Luginbuehl et al., 2004). Enhanced osteogenic differentiation and bone regeneration were observed in BMP-2-incorporated porous collagen scaffolds (Zhang et al., 2012). BMP-2-incorporated HAp-coated artificial ligament showed enhanced osseointegration and osteogenesis in bone tunnel (Jin et al., 2016). However, the conjugation between HAp and BMP-2 was intermediated by the electrostatic interaction (La et al., 2010), which may not sustain a long and stable release period of BMP-2. BMP-2 polypeptide has a short half-life and is easily inactivated *in vivo* alone (Wu et al., 2019), which may pose a negative impact on the effect of BMP-2 incorporation. Effective improvements are needed for BMP-2-immobilized surface modification.

Dopamine (DA), an adhesive protein that is derived from mussels, can be readily polymerized to form polydopamine (PDA)ad-layers by imine formation or Michael addition on the surface of various materials, such as polymers and bioceramics (Lee et al., 2009; Lynge et al., 2011; Wang and Stewart, 2013). Studies have proven that the PDA layer can intermediate the binding of bioactive molecules, such as protein, peptides, and DNA onto the scaffold surface (Lee et al., 2009; Zhou et al., 2010; Ren et al., 2011; Mrowczynski et al., 2013). The quinone/semiquinone formed by the catechol group on the PDA layer results in irreversible covalent conjugation between biomolecules and the surface of organic substitutes using similar Michael addition and Schiff formation reactions (Lee et al., 2007). Yao et al. (2020) found that bone cell differentiation and bone regeneration were enhanced in 3D-printed polylactic acid scaffolds with PDA modification and BMP-2 immobilization. PELA electrospun fibers immobilized with BMP-2 mediated by PDA showed better induction of differentiation into cartilage and bone in an acetabulum defect porcine model compared to the autotendon or PDA-coated PELA electrospun fibers groups (Wu et al., 2020). Previously, we have successfully incorporated mesoporous bioactive glass onto PET ligaments *via* PDA, and improved biocompatibility and bioactivity of PET ligaments were observed (Yu et al., 2017).

In this study, we aimed to immobilize BMP-2 onto PET ligaments mediated by PDA coating. Then, the influence of modified grafts on the attachment, proliferation, and osteogenic differentiation was tested by rat bone mesenchymal stem cells (rBMSCs). A graft-to-bone healing model was also established in rabbits to evaluate the efficacy of the modified PET ligaments.

## Material and Methods

### Preparation of PET Sheet Immobilized With BMP-2 Mediated by Polydopamine

PET sheets removed from a LARS ligament were immersed in 75% alcohol solution for 4 h to remove dirt. Subsequently, the sheet was washed with deionized water for three times and dried at 37°C for 24 h. The prepared sheets then were cut into discs with a 1-cm diameter for the following experiments.

Sheets with a 1-cm diameter were immersed in dopamine hydrochloride (Sigma-Aldrich, St. Louis, MO, United States) solution (2 mg/ml, 10 mM Tris-HCL buffer, and pH 8.5) and stirred at 160 rpm in an incubator for 6 h. Samples were thoroughly rinsed with ultrapure water and dried at 37°C overnight. The obtained scaffolds were named as PDA-PET. Polydopamine-coated PET sheets were immersed in BMP-2 (R&D Systems, Minneapolis, MN, United States) solution (250 and 500 ng/ml, 10 mM Tris-HCl buffer, and pH 8.5) and incubated at 37°C overnight. Finally, the prepared scaffolds were washed with ultrapure water and then dried in a drying oven at 37°C for 24 h. The obtained sheets were named as 250B-PDA-PET and 500B-PDA-PET respectively. The pure PET sheets were considered as control.

### Characterization of the Grafts

#### Scanning Electron Microscopy and Fourier-Transform Infrared Spectroscopy

The surface morphologies of all obtained scaffolds were examined by field-emission scanning electron microscope (FE-SEM) (Nova NanoSEM 450, FEI, United States) at a 20-kV accelerating voltage. The samples were vacuum coated with gold by sputtering prior to observation with SEM. The spectra of all grafts were tested using a Fourier transform infrared spectrometer (ATR-FTIR, Nicolet 6700, United States).

#### Hydrophilicity Properties

Static contact angle measurements were performed by a contact angle meter equipped with a high-resolution CCD camera (FM40Mk2 EasyDrop, Germany) to investigate the surface hydrophilicity of the PET, PDA-PET, 250B-PDA-PET, and 500B-PDA-PET grafts. Each sample was measured at three different locations (*n* = 6). A water drop of 3 μl in volume was placed on the surface of each graft. In that moment, images of droplets on the ligaments were captured using a side-view microscope connected to a camera (Nikon, United States). The contact angle was calculated by applying a spherical approximation using ImageJ 1.48 software.

#### Quantification of Immobilized BMP-2

Enzyme-linked immunosorbent assay (ELISA) was employed to measure the immobilized amount and long-term release kinetics of BMP-2. The PET sheets were treated with 500 μl of BMP-2 solution and incubated overnight at 37°C. After overnight incubation, supernatant was harvested for the measurement of remaining BMP-2 by an ELISA kit. Meanwhile, the amount of BMP-2 in the original solution (which are not treated with PET sheets) was determined using ELISA, and a difference in the values of the original solutions and the supernatant solutions was used to calculate the amount of immobilized BMP-2 on PET sheets. To investigate the long-term release of BMP-2, the sheets were immersed in 1 ml phosphate-buffered saline (PBS) and incubated at 37°C. At each predetermined time interval (1, 3, 5, 7, 14, 21, and 28 days), the supernatant was harvested and replaced with fresh PBS for continuous incubation until 28 days. The release of BMP-2 in all samples was quantified by an ELISA kit (R&D Systems, Minneapolis, MN, United States), and the results were then expressed according to previous studies (Cho et al., 2014).

### *In Vitro* Experiments

#### Cell Culture

Bone mesenchymal stem cells derived from Sprague–Dawley rats (rBMSCs) were used to examine the cytocompatibility and bioactivity of the modified PET sheets in this study. rBMSCs were cultured at 37°C in a humidified incubator with 5% CO_2_ in MEM alpha medium (α-MEM) (HyClone, United States) containing 10% fetal bovine serum (Gibco, United States) and 100 U/ml penicillin–streptomycin (Sigma), and the medium was replaced every other day. The passage four to five (P4 to P5) rBMSCs was used for the following tests.

#### Cell Proliferation and Attachment

All grafts were sterilized by ethylene oxide gas before experiment. Thereafter, the scaffolds were transferred into a non-treated 24-well plate. rBMSCs in the logarithmic phase were seeded onto each sheet at a density of 1 × 10^4^ cell.

Cell proliferation was assessed at 1, 3, and 7 days using the Cell Counting Kit-8 (CCK-8, Dojindo, Japan) in this study. At each predetermined time point, the culture medium was removed and the samples were washed with PBS thrice; 360 μl α-MEM medium and 40 μl CCK-8 solution were added into each well. Samples were fully incubated for 4 h at 37°C, and 100 μl of supernatant for each sample was transferred into a non-treated 96-well plate, and absorbance at 450 nm was measured using a microplate reader (TECAN Infinite 200 PRO, Switzerland). We observed cell morphology and adhesion on scaffolds after 5 days of culturing *via* scanning electron microscopy (SEM, FEI Quanta 450). After a scheduled interval, samples were gently rinsed with PBS, fixed in 2.5% glutaral solution at 4°C overnight, washed thrice with ddH_2_O, dehydrated in ethanol series (30%, 50%, 70%, 90%, 95%, and 100%; 10 min each, twice), and dried at room temperature. The specimens were gold coated for examination.

#### Alkaline Phosphatase Assay

Alkaline phosphatase (ALP) activity was determined using an ALP assay kit (Cat. No. P0321, Beyotime). Briefly, rBMSCs were seeded on each sheet at a density of 1 × 10^5^and then treated with an osteogenic induction medium (10 mM β-glycerophosphate, 50 μM ascorbic acid-2-phosphate, and 100 nM dexamethasone in complete medium) for 7 or 14 days. Samples were washed three times with PBS and dissolved *via* RIPA lysis buffer. Afterwards, the harvested solution was centrifuged at 12,500×*g* for 5 min. Finally, supernatant was collected for ALP activity assay according to the manufacturer’s instructions. ALP activity was normalized to the total protein content.

#### Osteogenesis-Related Gene Expression Analysis

The expression level of osteogenic genes in rBMSCs was examined by RT-PCR. Cells seeded on the sheets were incubated in osteoinductive medium for 7 or 14 days, and total RNA was extracted from the rBMSCs using an RNA extraction kit (Cat. No. AG21017 Accurate Biology, China). Thereafter, the harvested total RNA was reverse-transcribed into complementary DNA (cDNA) using Evo M-MLV RT Kit with gDNA Clean for qPCR II (Cat. No. AG11711, Accurate Biology, China). Osteoblast differentiation-related genes, osteopontin (OPN), runt-related transcription factor 2 (Runx 2), osteocalcin (OCN), and ALP were tested in this study. Gene amplification was performed in the real-time PCR instrument (ABI 7300) using Pro Taq HS Premix Probe qPCR Kit (Cat. No. AG11704, Accurate Biology, China). Glyceraldehyde 3-phosphate dehydrogenase (GAPDH) was regarded as the internal reference gene. Each sample was assayed in triplicate.

#### Alizarin Red S Staining and Quantitative Analysis of Mineralized Bone Nodules

After incubation in osteogenic induction medium for 21 days, rBMSCs were rinsed in PBS thrice and fixed in 4% paraformaldehyde for 10 min. Then, 0.5% Alizarin Red S (pH 4.3, Sigma) was reacted with each sample at room temperature for 30 min and washed with ddH_2_O until water was clean. Then, mineralized nodule was observed under a microscope. The amount of mineralized nodule was quantified as well. The stained samples were treated with 10% (*w*/*v*) cetylpyridinium chloride for 1 h at RT, and the absorbance at 572 nm was measured with a microplate reader (TECAN Infinite 200 PRO, Switzerland).

### Animal Experiments

#### Graft Implantation Into Rabbits

All animal experimental procedures were approved by the Laboratory Animal Welfare and Ethics Committee at our institution. Fifteen mature male New Zealand rabbits (male, 10 weeks old, 2.7 ± 0.3 kg) were randomly assigned to three different groups, named as the PET group, PDA-PET group, and 500B-PDA-PET group. An extra-articular graft-to-bone healing procedure was operated on each rabbit bilaterally. The rabbits were anesthetized with 3% (*w*/*v*) pentobarbital (30 mg/kg) *via* an intraperitoneal injection. Grafts, with a length of 2.0 cm and a diameter of 2.0 mm, were implanted into tunnels drilled by a 2-mm-diameter Kirschner wire through the condyle of the femur. Postoperatively, penicillin (50 KU/kg) were administered through continuous injections for 3 days. The rabbits were sacrificed at 12 weeks after operation for the following tests.

#### Mechanical Tests

Five freshly harvested femurs were prepared for mechanical tests. A No. 5 WilSuture Poly suture, which is extended out of the drilling tunnel entrance, was used to suture the implanted graft. The sample was mounted onto a special jig, and it was made sure that the tension on each graft was in accordance with the pullout test axis. The load-to-failure test was conducted with a Material Testing System (Instron, United States) at an elongation rate of 2 mm/min. The tensile load was recorded to calculate the ultimate failure load (N).

#### Micro-Computer Tomography Examination

The harvested femur condyles were fixed in 4% paraformaldehyde for 2 weeks. The samples were scanned by micro-CT (SkyScan 1176, Kontich, Belgium) at 18-μm resolution. Along the longitudinal axis of the femur bone tunnel, a 2 × 10 mm^2^ cylindrical region of interest (ROI) was reconstructed from the middle part of the tunnel for each sample. Three-dimensional images were obtained using the 3-D Creator software, and data were analyzed by VGStudio MAX (Volume Graphics, Germany) software. Microstructural parameters such as the bone volume/total volume (BV/TV, %) and bone surface/bone volume (BS/BV, 1/mm) were determined.

#### Histological Analysis

The femur specimens were fixed in 4% paraformaldehyde for 48 h, decalcified in 5% nitric acid for 2 weeks, and dehydrated in gradient ethanol. Subsequently, the dehydrated specimens were embedded in paraffin for sectioning and staining. Sections were perpendicular to the longitudinal axis of the bone tunnel with a thickness of 5 μm. The slices were stained with hematoxylin and eosin (HE) staining reagent for evaluation. The stained slices were scanned by a histology digital scanning system (NanoZoomer S210, Hamamatsu), and pictures were obtained *via* NDP.view 2 software. Finally, the evaluation of the graft-to-bone healing was conducted with staining images.

### Statistical Analysis

The results were expressed as the mean ± standard deviation (SD). Data were analyzed using SPSS 20.0 and GraphPad Prism 10 software. One-way ANOVA and Student’s *t*-tests were used to determine the level of significance, and *p* value less than 0.05 was considered as statistically significant.

## Results

### Characterizations

**FIGURE 1:**
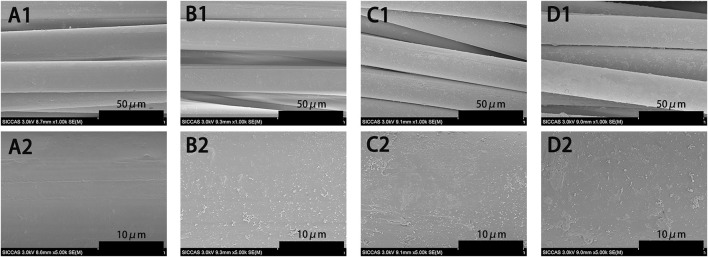
SEM images of pure PET **(A1, A2)**, PDA-PET **(B1, B2)**, 250B-PDA-PET **(C1, C2)**, and 500B-PDA-PET **(D1, D2)**. SEM, scanning electron microscopy; PET, polyethylene terephthalate; PDA, polydopamine

**FIGURE 2:**
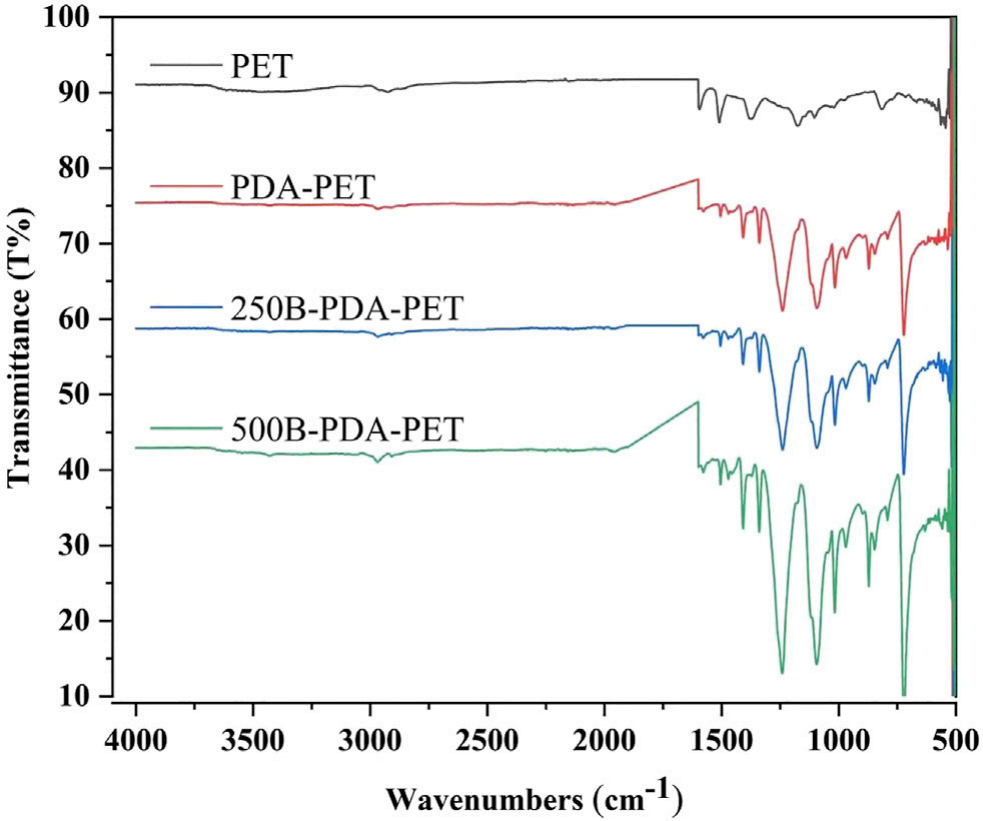
Fourier-transform infrared spectroscopy (FTIR) of PET, PDA-PET, 250B-PDA-PET, and 500B-PDA-PET.

We examined the morphology of PET, PDA-PET, 250B-PDA-PET, and 500B-PDA-PET samples *via* SEM. As is shown in Figure 1, the pure PET (Figure 1 A1, A2) fiber has a smooth surface without any attachment. A layer with fine coarse particles was observed on the surface of the PDA-PET group (Figure 1 B1, B2). The 250B-PDA-PET (Figure 1 C1, C2) and 500B-PDA-PET (Figure 1 D1, D2) also showed a coating with fewer particles than that on the PET group, and the layer modified by BMP-2 seemed thicker than the PDA layer in some areas. For the PDA-PET scaffold, the peaks at 1,506 cm^−1^ (the indole structure of PDA) and 1,281 cm^−1^ (the stretching vibration of catechol hydroxyl) prove the successful coating of PDA. The element component of BMP-2 was identical to that of PDA; therefore, the characteristic absorption peaks in 250B/500B-PDA-PET grafts are similar to those in the PDA-PET group (Figure 2, FTIR results).

**FIGURE 3:**
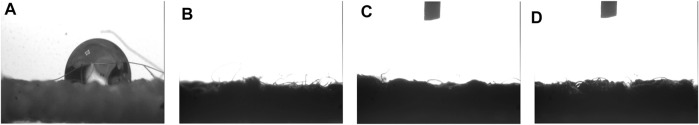
Water contact angle of PET **(A)**, PDA-PET **(B)**, 250B-PDA-PET **(C)**, and 500B-PDA-PET **(D)**.

The water contact angle was also examined to investigate changes in hydrophilicity on the scaffold surface (Figure 3). The PET grafts exhibited a static water contact angle of 90.9° ± 5.0° after a measurement time period of 10 s. In the PDA coating or BMP-2-immobilized groups, the water drop penetrated into the surface so fast that a static water contact angle cannot be measured, which demonstrated a better wettability compared to the PET group.

### Quantification and Release of Immobilized BMP-2

**FIGURE 4:**
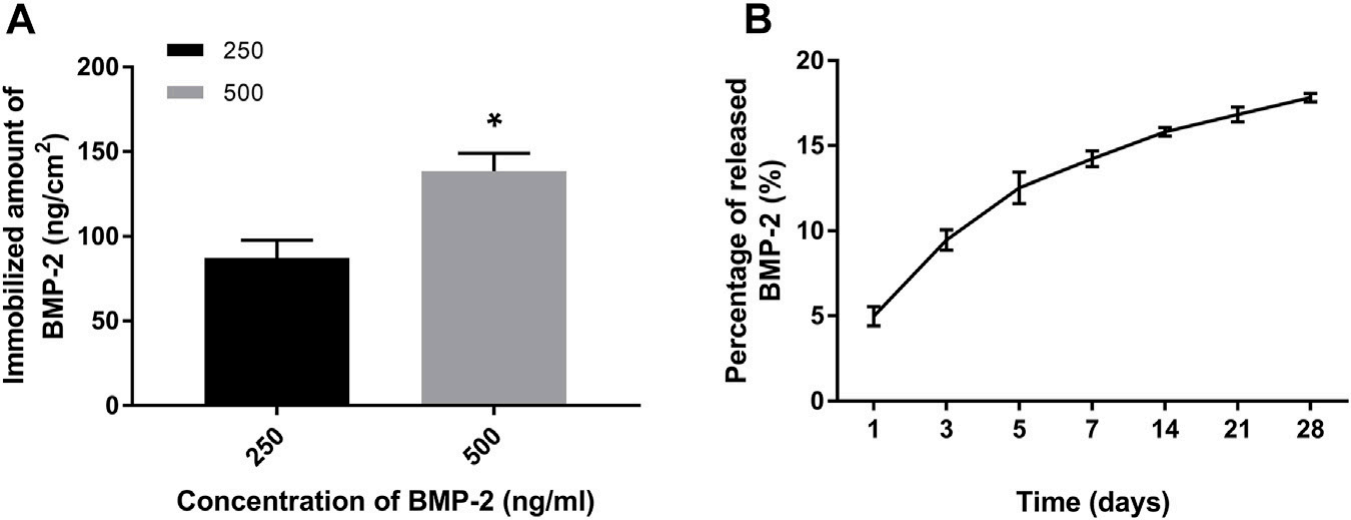
Quantification and release of immobilized BMP-2 on polydopamine-coated PET using ELISA. **(A)** The amount of immobilized BMP-2 on PET fabricated using different BMP-2 concentrations. Asterisk (*) indicates a significant difference compared to the 250 ng/ml immobilized BMP-2 group, *p* < 0.05. **(B)** The dynamic changes of released BMP-2 from the PET during a 28-day period in phosphate-buffered saline (PBS) (37°C).

The amount of immobilized BMP-2 on PDA-coated grafts was measured indirectly (Lee et al., 2012). We found that the amount of BMP-2 increased as the concentration of BMP-2 treatment solutions increased (Figure 4A). The grafts treated with 500 ng/ml of BMP-2 showed more immobilized BMP-2 (138.4 ± 10.6 ng/cm^2^) compared with those treated with 250 ng/ml (83.1 ± 9.5 ng/cm^2^). We then investigated the dynamic release of BMP-2 on PDA-coated scaffolds for up to 28 days. Only the 500B-PDA-PET group was examined. At the initial 7 days, around 14% of immobilized BMP-2 were released, which is nearly five times the release amount during day 7 to day 28. At the end of the 28-day period, the results showed that more than 80% of the immobilized BMP-2 was retained on the surface of grafts (Figure 4B).

### Cell Attachment and Proliferation

**FIGURE 5:**
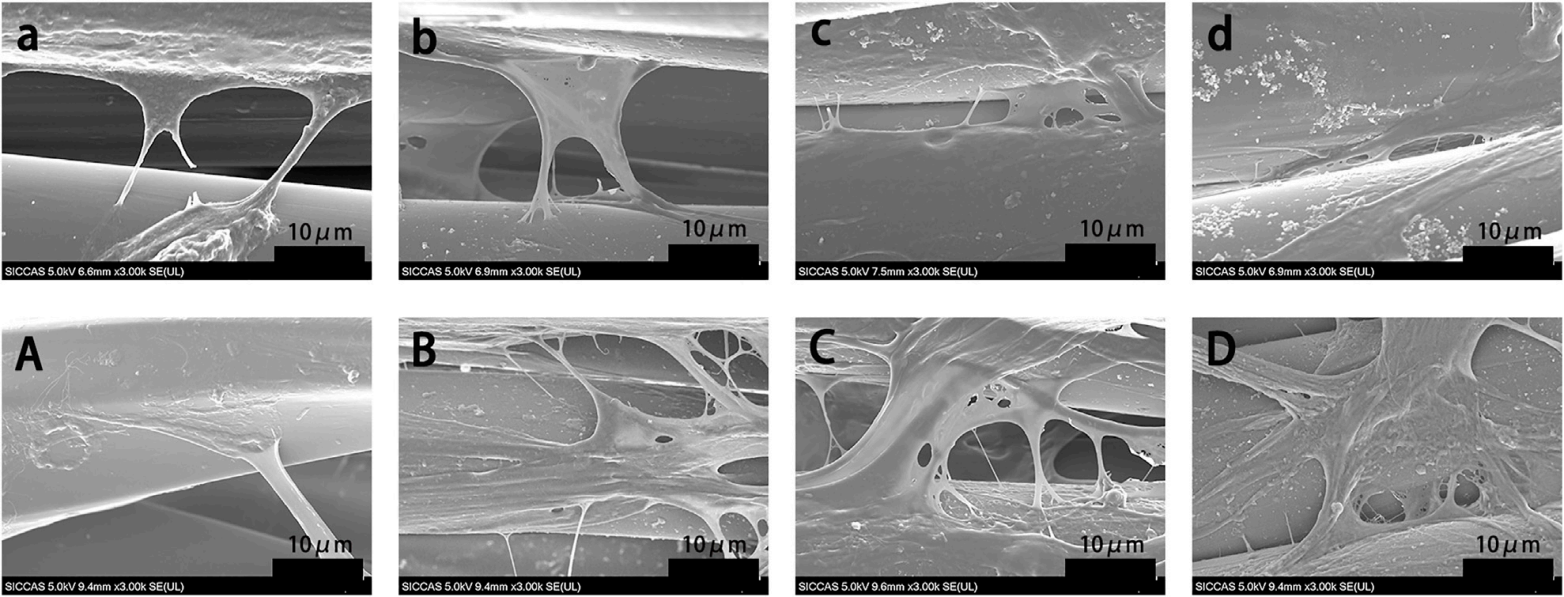
SEM images of rat bone mesenchymal stem cells (rBMSCs) cultured on PET **(a, A)**, PDA-PET **(b, B)**, 250B-PDA-PET **(c, C)**, and 500B-PDA-PET **(d, D)** for 1 day **(a–d)** and 5 days **(A–D)**.

**FIGURE 6:**
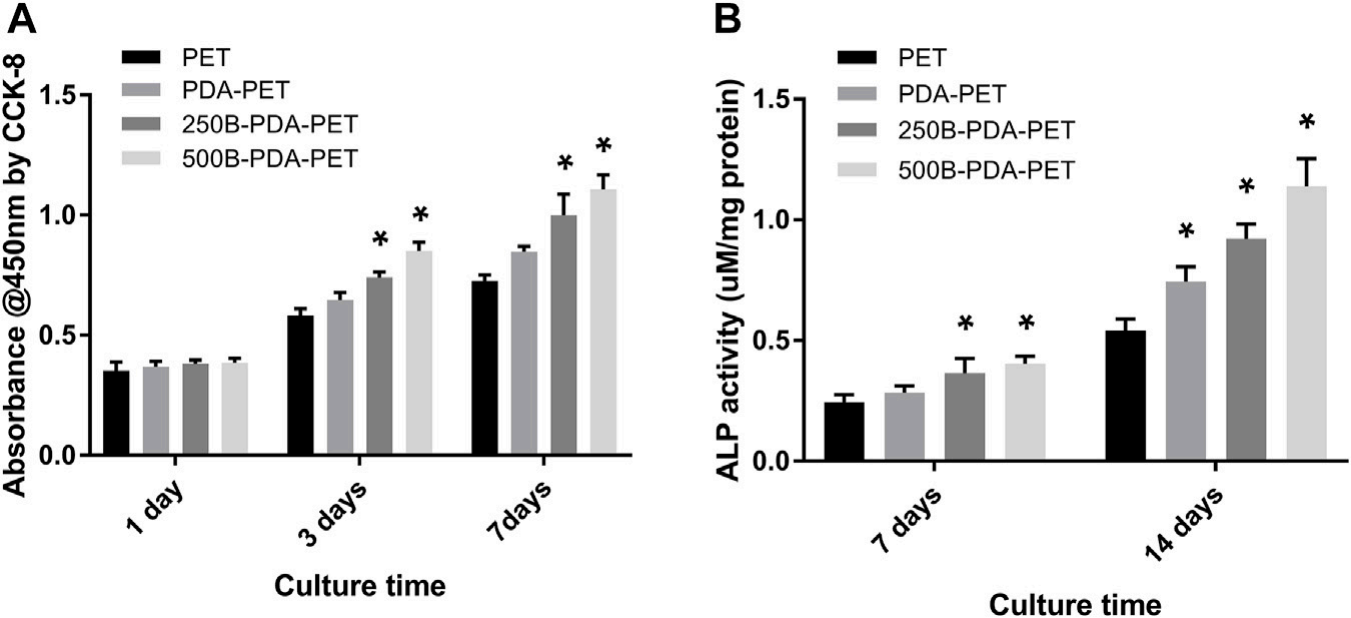
**(A)** The proliferation of rBMSCs incubated on different groups of grafts at 1, 3, and 7 days using CCK-8. **(B)** Alkaline phosphatase (ALP) activity assay of rBMSCs cultured in an osteogenic induction solution for 7 and 14 days. Asterisk (*) indicates a significant difference compared to the PET group, *p* < 0.05.

The morphology of rBMSCs cultured on grafts for 1 and 5 days was evaluated using SEM (Figure 5). More rBMSCs were observed on the surface of the BMP-2-immobilized grafts than in other groups. Cells seeded on the PDA-PET, 250B-PDA-PET, as well as the 500B-PDA-PET showed multiple cellular morphologies such as pseudopods and lamellipodia. The proliferation of rBMSCs incubated on the grafts for 1, 3, and 7 days was analyzed using CCK-8 (Figure 6A). Optical density (OD) values in the 250B-PDA-PET or 500B-PDA-PET group were significantly different compared with the PET group at 3 and 7 days culturing time points (*p* < 0.05).

### Cell Differentiation *In Vitro*

**FIGURE 7:**
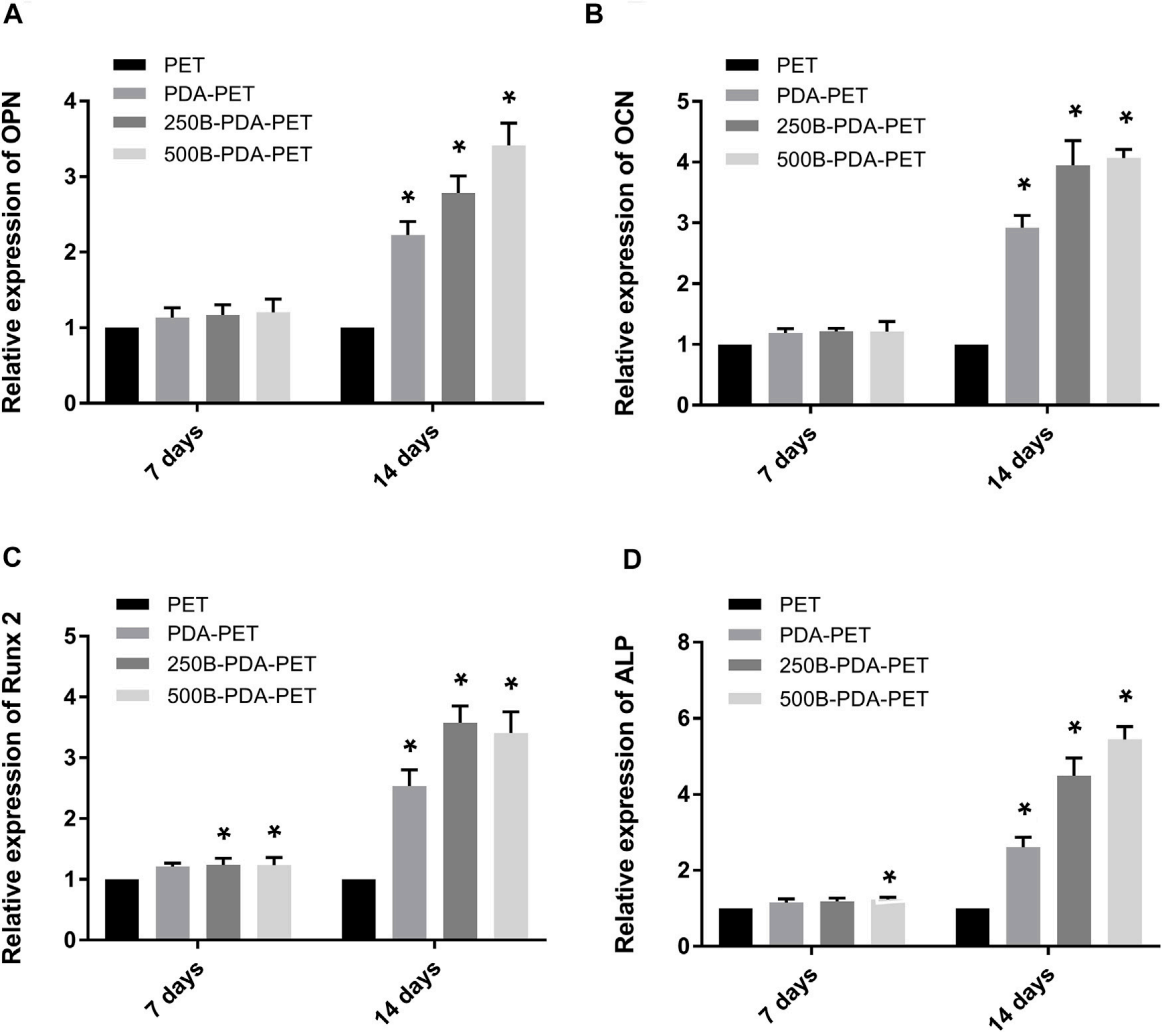
Expression level of **(A)** osteopontin (OPN), **(B)** osteocalcin (OCN), **(C)** runt-related transcription factor 2 (Runx 2), and **(D)** ALP of rBMSCs cultured on different groups of grafts using RT-PCR. Asterisk (*) indicates a significant difference compared to the PET group, *p* < 0.05.

To investigate the impact of BMP-2-immobilized grafts on the osteogenic differentiation of rBMSCs, we measured ALP activity and the expression levels of osteoblast-specific genes, including OCN, OPN, ALP, and Runx 2, at 7 or 14 days (Figure 7). The ALP activity of rBMSCs in the 500B-PDA-PET group was significantly higher than that in other groups at each predetermined time point (*p* < 0.05) (Figure 6B). The transcription levels of osteogenesis-related genes, including OPN, OCN, Runx2, and ALP, were analyzed *via* RT-PCR. The results demonstrated that the expression level of these osteogenic markers in the PDA-PET or 250B-PDA-PET or 500B-PDA-PET group was significantly higher than that in the PET group at 14 days (*p* < 0.05), and the 500B-PDA-PET group showed the highest expression level.

**FIGURE 8:**
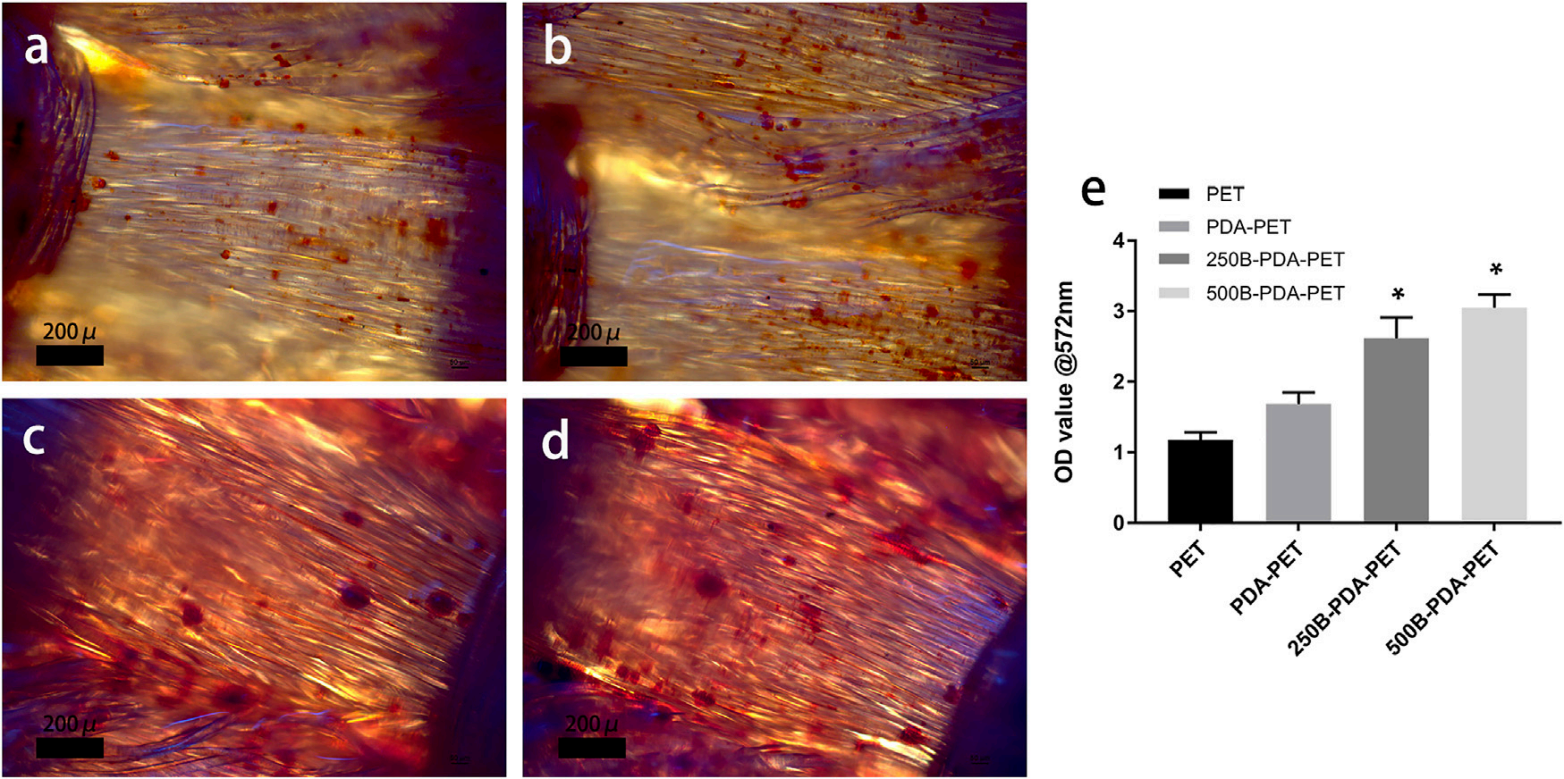
**(A)** Images of mineralized calcium nodules after 21 days of osteogenic induction using Alizarin Red S staining. **(B)** Quantification of mineralized calcium nodules. Asterisk (*) indicates a significant difference compared to the PET group, *p* < 0.05.

After 21 days of induction, samples were stained using Alizarin Red S, and quantification was performed. As is shown in Figure 8, calcium nodule sedimentation was observed on the surface of different grafts. Calcium nodules on the surface of the BMP-2-immobilized group were larger than those on the surface of the PDA-PET or PET group. Quantification of the calcium nodules (Figure 8B) showed that there was significantly higher calcification in the 500B-PDA-PET group (OD value 3.91 ± 0.15) than in other groups (*p* < 0.05). These results are consistent with the ALP activity assay.

### Mechanical Tests

**FIGURE 9:**
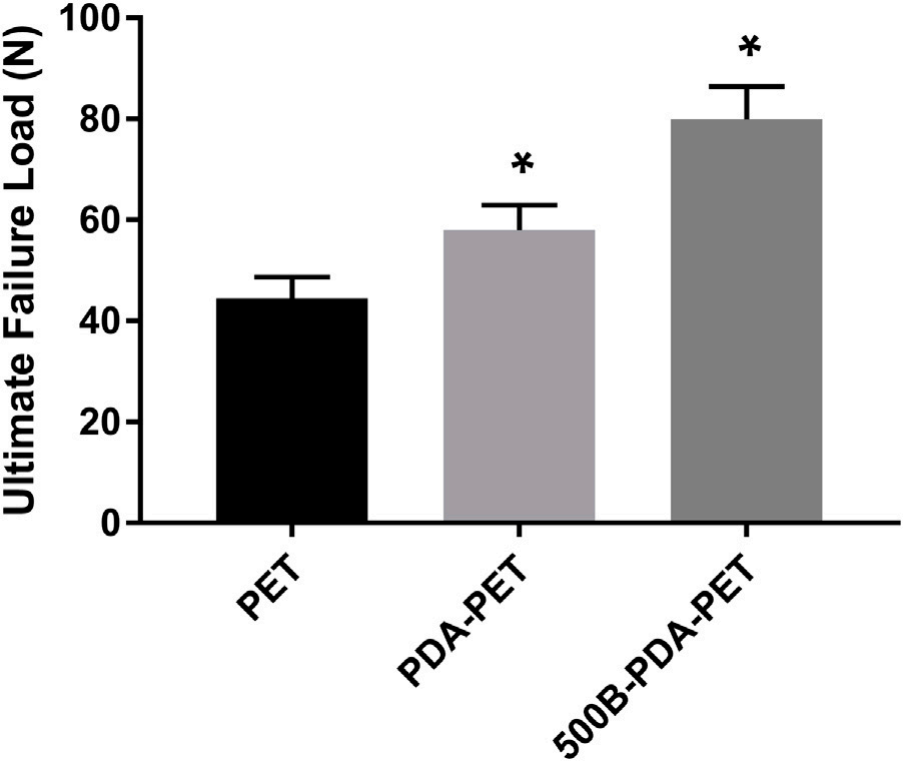
The ultimate failure load of grafts after implantation of 8 weeks. Asterisk (*) indicates a significant difference compared to the PET group, *p* < 0.05.

An extra-articular graft-to-bone healing animal model was used to evaluate the osseointegration of the graft within the host bone. At 8 weeks after implantation, the ultimate failure load of the 500B-PDA-PET group was 79.93 ± 6.49 N and was significantly higher than that of the PET and PDA-PET groups, with an ultimate failure load at 44.25 ± 4.01 N and 58.03 ± 4.91 N, respectively (*p* < 0.05) (Figure 9).

### Micro-CT for New Bone Formation

**FIGURE 10:**
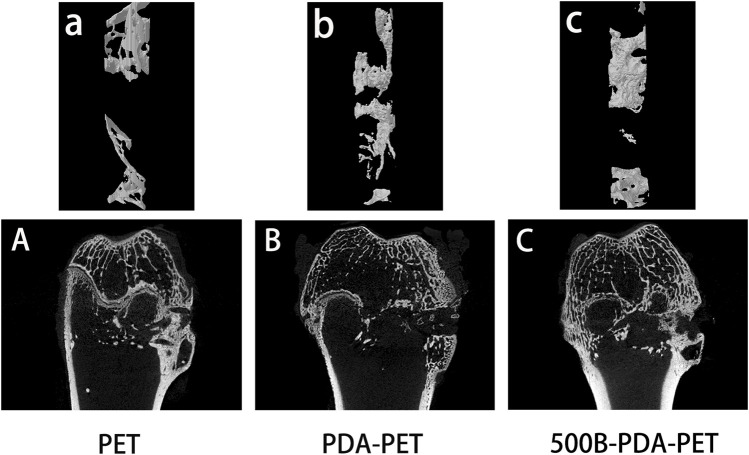
3D) reconstruction and 2D images of grafts after implantation of 8 weeks.

**FIGURE 11:**
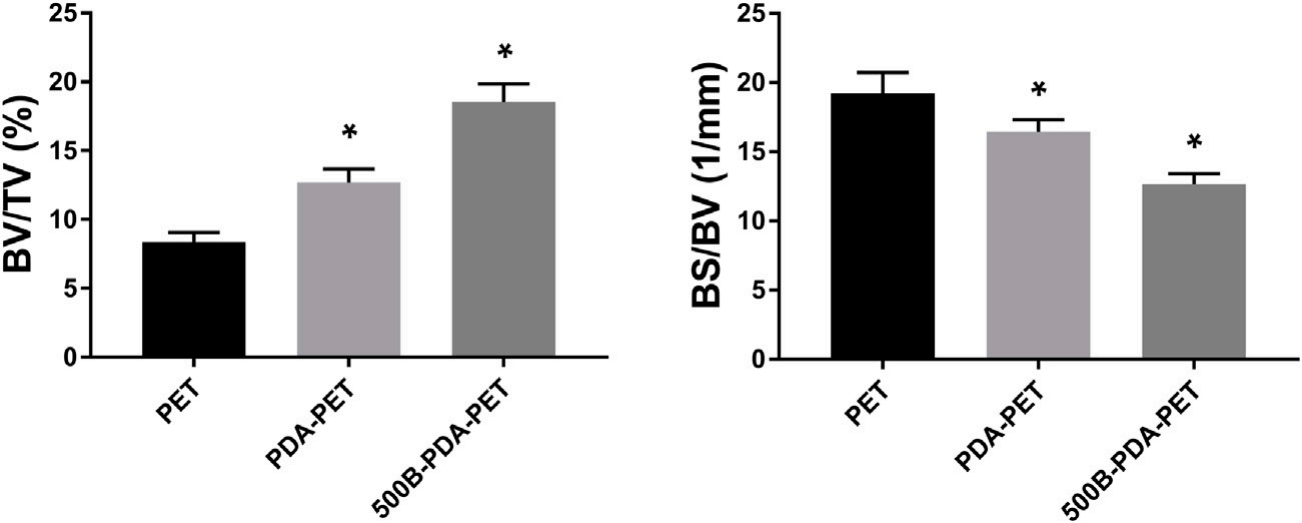
Analysis of new bone formation in bone tunnels after implantation of 8 weeks. BV/TV indicates the bone volume/total volume, and BS/BV indicates the bone surface/bone volume. Asterisk (*) indicates a significant difference compared to the PET group, *p* < 0.05.

The harvested femurs were examined by micro-CT for the evaluation of bone regeneration. Identically sized ROIs around the grafts were reconstructed. Two- and three-dimensional images of reconstruction are presented in Figure 10. We found that there was more bone formation around and inside the grafts in the 500B-PDA-PET group than in the other two groups. Microstructural parameter analysis demonstrated that the BV/TV in the 500B-PDA group was significantly higher than those in the PET or PDA-PET group (*p* < 0.05). On the contrary, significant decreases in BS/BV were observed (*p* < 0.05) (Figure 11).

### Histological Analysis

**FIGURE 12:**
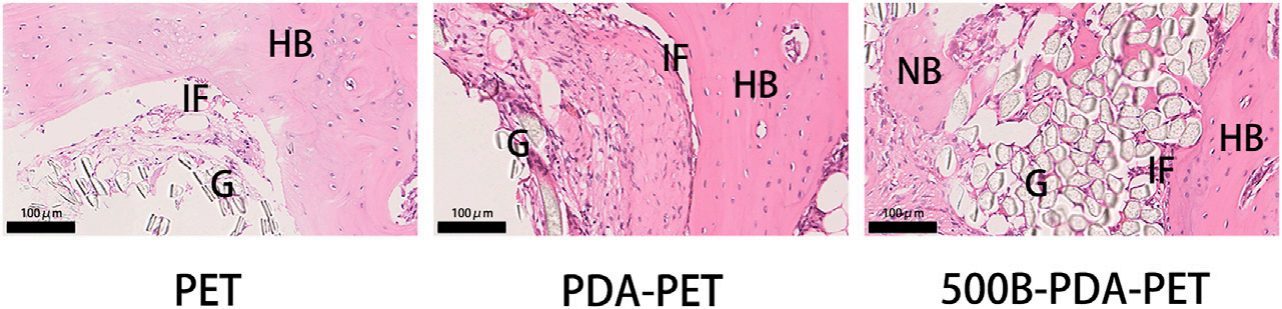
HE) staining of grafts sectioned perpendicularly to the longitudinal axis at 8 weeks. G, graft; HB, host bone; IF, interface; NB, new bone.

Histological staining was performed for the evaluation of graft-to-bone healing effect. As the pictures of HE staining show, grafts in the bone tunnel bonded with the host bone by relatively loose fibrous tissue, and almost no visible new bone was formed in the PET group. In the PDA-PET group, the interface between graft and native bone was denser than the PET group, which presented a trend for osteogenic differentiation. In the 500B-PDA-PET group, a tight connection was formed between the scaffolds and host bone with a certain quantity of new bone regeneration. For the PDA-PET group, some new bone formed in the interface, and it seemed to be unmatured (Figure 12).

## Discussion

The PET ligament is one of the most commonly used artificial grafts in ACL reconstruction, and favorable feedbacks were received for long-term follow-ups. However, the inferior biocompatibility and bioactivity of the PET grafts might result in the enlarged tunnels and failure to reconstruction (Yu et al., 2017). In this study, we successfully ameliorated the surface properties of PET ligaments by the immobilization of BMP-2 *via* PDA coating. Our results proved that the modified PET ligaments could enhance the attachment, proliferation, and osteogenic differentiation of rBMSCs and promote the tight bonding between the grafts and native bone *in vivo*, which was partially attributed to the continuous and stable release of BMP-2.

The characterizations of modified grafts were examined. SEM showed that the surface of PDA-coated grafts as well as BMP-2-immobilized grafts presented a layer with rough particles while the PET grafts presented a smooth surface. In addition, difference of the surface components was observed among groups *via* FTIR. These results combined suggested the successful fabrication of PET grafts with PDA coating and BMP-2 immobilization.

As one of the bone morphogenetic protein families, BMP-2 is widely applied in orthopedic surgeries such as open tibial fractures, articular cartilage damage, non-unions, and lumbar spine fusion (Poon et al., 2016). BMP-2-mediated surface modification is beneficial for the successful osseointegration between implants and native bone. Studies showed that BMP-2 was able to promote cell chemotaxis, proliferation, and osteogenic differentiation (Ribeiro et al., 2015) and presented favorable osteoinductive property for bone growth and regeneration (Poon et al., 2016). In light of its excellent bioactivity, BMP-2 has great potential to be developed in the application of surface modification. However, several issues in the immobilization of BMP-2 also trouble the researchers. For example, denaturation and inactivation of BMP-2 easily occur under physiological conditions and when applied alone (Takahashi et al., 2005; Wu et al., 2019). PDA, a polymerized form of dopamine, resulted from the interaction between catechol and amine groups in dopamine in a slightly alkaline solution (Lee et al., 2007). It is an attractive candidate for tissue engineering as articles reported, especially in surface modification. PDA can easily be deposited onto the surface of various grafts to form an ad-layer for bonding with diverse bioactive substances and peptides *via* imine formation or Michael addition (Lee et al., 2009; Ku et al., 2010; Lynge et al., 2011; Chien et al., 2012). The measurement of a water contact angle showed that surface hydrophilicity was greatly ameliorated by PDA coating. However, the water drop penetrated into the coating so fast that a static water contact angle cannot be measured and the difference of static water contact angle between the PET and PDA-PET groups cannot be calculated as well. The enhanced hydrophilicity was beneficial for cell adhesion, thus triggering the intracellular signal pathway and promoting cell proliferation (Kao et al., 2015).

BMP-2 presented a favorable ability to enhance bone healing; however, potential complications also existed, especially off-label use of BMPs (Boden, 2005). The most common complication is extra bone formation, which is called heterotopic ossification (Boraiah et al., 2009). Researchers also found that a high dose (>40 mg) of rhBMP-2 administration is associated with the increased risk of cancer (Dimar et al., 2009). Therefore, we chose the solution at a concentration of 250 and 500 ng/ml, a relatively low dose, to immobilize BMP-2 onto the grafts. Finally, the amounts of BMP-2 immobilized on the graft were 83.1 ± 9.5 ng/cm^2^ and 138.4 ± 10.6 ng/cm^2^, respectively. During a 28-day dynamic release period, more than 80% of the immobilized BMP-2 was retained. These results are in accordance with previous reports. Cho et al. (2014) found that immobilized BMP-2 on polydopamine-coated PLLA nanofibers showed approximately 90% retention efficiency over 28 days. A hydroxyapatite coating was also used to mediate the immobilization of BMP-2 on a titanium alloy, and a quarter of the immobilized amount of BMP-2 was released during the first 7 days (Cai et al., 2014). Our results showed a stable and continuous delivery of BMP-2 of the modified grafts for a relatively long period, which is favorable for bone regeneration and may avoid dose-related complications *in vivo*. The sustainable release of BMP-2 may lie in the tightly covalent binding between the catechol and quinone groups on PDA and the amino side chains of BMP-2 (Chien and Tsai, 2013).

In cellular experiments, CCK-8 assay showed that OD values in all groups increased with culture time, and the 500B-PDA-PET grafts exhibited a higher OD at 3 and 7 days of culturing than the other groups. More attached cells on the surface of the BMP-2-immobilized group were detected than on the PET group *via* SEM. These results indicated that BMP-2-modified scaffolds could promote the attachment and proliferation of rBMSCs and exhibited better biocompatibility. The ALP activity assay and examination of the transcription levels of osteogenesis-related genes confirmed the improved osteogenic differentiation after PDA coating and BMP-2 immobilization. In addition, the mineralization of the grafts was evaluated *via* Alizarin Red S staining. Larger and more calcium nodules were observed in the 500B-PDA group than in the other three groups, and quantification indicated a significant difference. These results indicated that the immobilization of BMP-2 mediated by PDA coating posed a favorable impact on the differentiation of rBMSCs.

Previous studies detected that a loose granulation fibrous layer was formed between the grafts and host bone (He et al., 2012; Cho et al., 2013; Jiang et al., 2014). And, this might be the main reason for the enlarged bone tunnels, unstable knee joints, or the failure of ACL reconstruction. Our *in vivo* results demonstrated that the ultimate failure load in the 500B-PDA group is significantly higher than that in the PET or PDA-PET group. The micro-CT examination showed a synergic effect of the BMP-2 and PDA on the osseointegration and bone regeneration. BV/TV in the PDA group was significantly higher compared with the PET group, and BV/TV in the 500B-PDA-PET group was significantly higher than that in the PDA-PET group. However, the changes of BS/BV presented an opposite trend. Consistent with the biomechanical tests, the histological staining showed a tight binding between the BMP-2-immobilized grafts and the native bone, indicating an improved graft-to-bone healing. All these results demonstrated that the BMP-2 and PDA-modified PET artificial ligaments exhibited an enhanced biocompatibility, bioactivity, and osseointegration. However, the specific mechanism cannot be clarified currently, and further investigations need to be performed.

## Conclusion

In this study, we prepared the PET artificial ligaments immobilized with BMP-2 *via* polydopamine coating. The immobilization of BMP-2 mediated by polydopamine coating on PET artificial ligament surface could enhance the compatibility and bioactivity of the scaffolds and the graft-to-bone healing *in vivo*, which is beneficial for the wide application of PET ligaments.

## Data Availability

The raw data supporting the conclusions of this article will be made available by the authors, without undue reservation.
